# Programmed cell death 1 blockade in the setting of severe ocular sarcoidosis: Cancer immunotherapy in a patient with autoimmunity

**DOI:** 10.1016/j.jdcr.2025.04.017

**Published:** 2025-04-26

**Authors:** Charlotte Read, Shailender Bhatia, Mariam Totonchy

**Affiliations:** a Department of Dermatology, University of Washington, Seattle, Washington; b Department of Medicine, Imperial College London, London, UK; c University of Washington/Fred Hutch Cancer Center, Seattle, Washington; d Skin Surgery Center, Bellevue, Washington

**Keywords:** autoimmune disease, cutaneous squamous cell carcinoma, immunotherapy, ocular sarcoidosis, PD-1 inhibitor

## Introduction

Immune checkpoint inhibitors (ICIs), such as inhibitors of the programmed cell death 1 (PD-1) pathway, have revolutionized the field of cutaneous oncology. Over the last few years, anti–PD-1 agents including cemiplimab and pembrolizumab have become the standard systemic therapy for patients with locally advanced or metastatic cutaneous squamous cell carcinoma (cSCC) that is considered incurable with surgery or radiation alone.^1^ However, the use of ICI is relatively contraindicated in patients with prior organ transplant or those with pre-existing autoimmune disease (AID) due to high risk of toxicities, including allograft rejection or exacerbation of underlying AID, respectively. In this case report, we highlight the successful use of pembrolizumab in a patient with metastatic cSCC arising in the setting of prolonged immunosuppression due to severe ocular sarcoidosis.

## Case report

**Fig 1:**
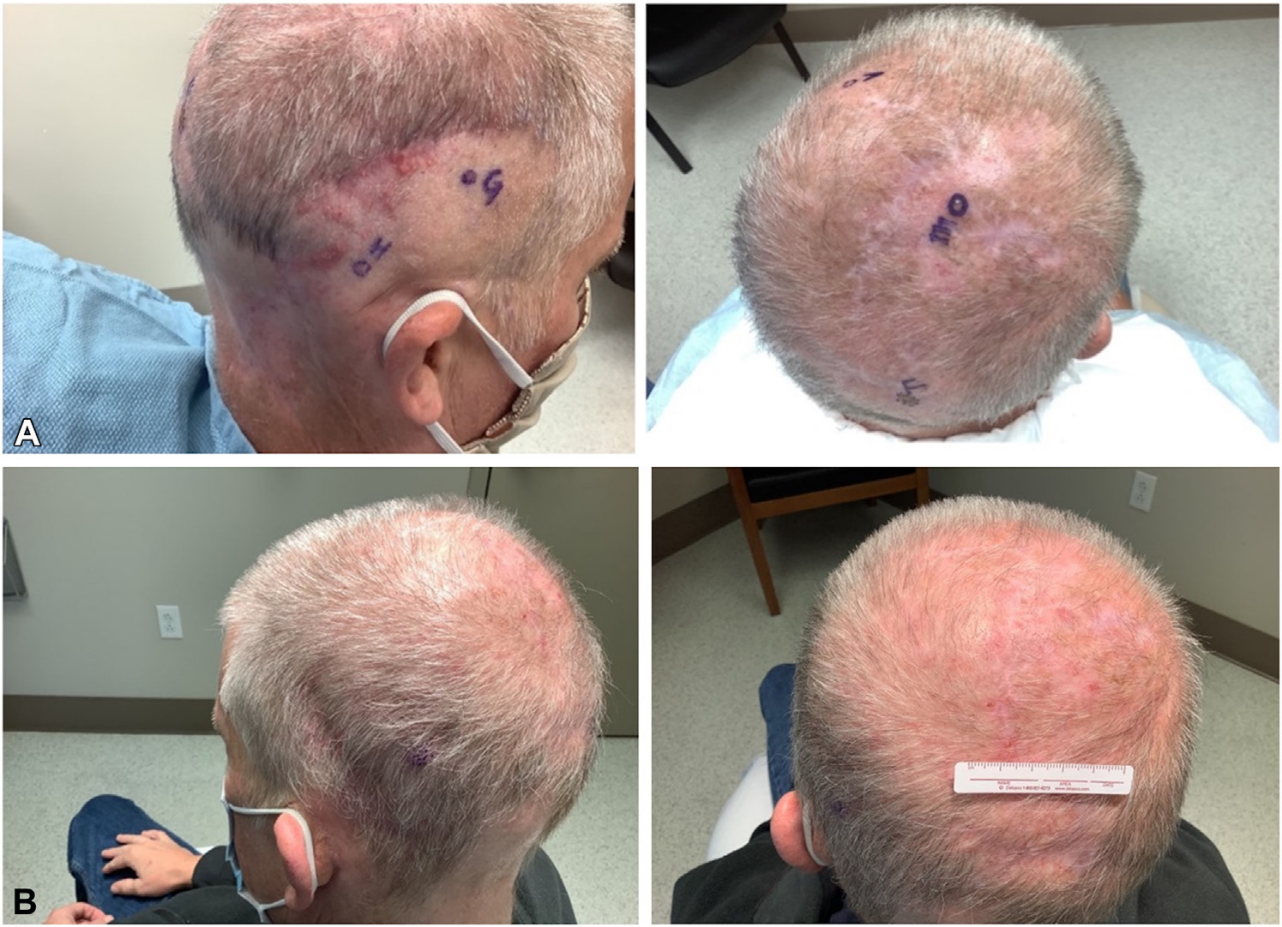
Comparison of clinical presentation pretreatment and post-treatment with pembrolizumab (initiated in September 2022) in a case of metastatic cutaneous squamous cell carcinoma. **A,** Clinical presentation in 2021: multiple cSCC metastases noted on the scalp. **B,** Clinical presentation in February 2023, 5 months after initiating pembrolizumab: regression of scalp metastases. *cSCC*, Cutaneous squamous cell carcinoma.

**Fig 2:**
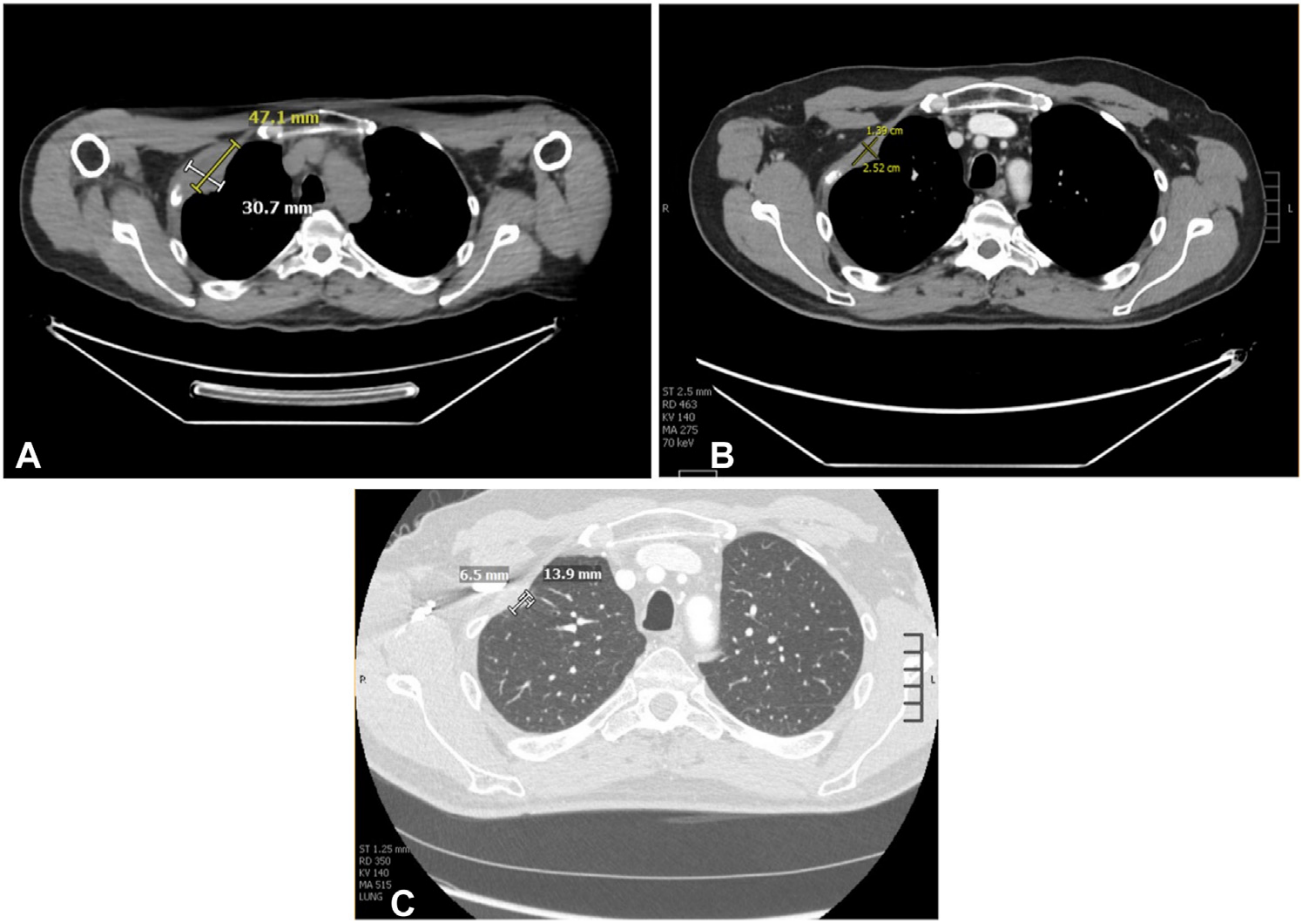
Radiographic presentation of right upper chest wall mass pretreatment and post-treatment with pembrolizumab (initiated in September 2022) in a case of metastatic cutaneous squamous cell carcinoma. **A,** Represents imaging 1 month prior to treatment with pembrolizumab, (**B**) represents imaging 6 months into treatment with pembrolizumab, and (**C**) represents imaging 18 months into treatment with pembrolizumab. **A,** Radiographic presentation of right upper chest wall mass measuring 4.7 × 3.0 cm in August 2022. **B,** Radiographic presentation of right upper chest wall mass measuring 1.4 × 2.5 cm in January 2023. **C,** Radiographic presentation of right upper chest wall mass measuring 0.65 × 1.39 cm in January 2024.

A 62-year-old White male developed multiple in-transit metastases of cSCC in 2020, 6 years after removal of the primary infiltrative cSCC on the scalp in 2014. This occurred in the setting of long-term systemic immunosuppression with mycophenolate mofetil (1.5 g/day, from 2014 to 2020) and intermittent courses of high-dose prednisone (up to 40 mg/day) for the treatment of panuveitis due to ocular sarcoidosis. After diagnosis of recurrent cSCC in 2020, his immunosuppressive regimen was weaned down to only prednisone 10 mg every other day. Over the next 2 years, he was treated with several Mohs procedures for multifocal in-transit metastases, and bilateral cervical lymph node dissections for metastases in the right suboccipital lymph node and contralateral left cervical lymph node metastases. He also received courses of radiation therapy to bilateral scalp and neck regions. Unfortunately, he continued to have additional loco-regional recurrences in the head and neck region as well as distant metastases including a right apical chest wall mass. In September 2022, the patient was started on pembrolizumab 200 mg intravenous every 3 weeks following extensive discussion of the risks and benefits given his underlying ocular sarcoidosis (Fig 1). 4 months later, computed tomography imaging studies showed complete regression of the scalp and cervical lymph node disease and partial regression of the lung metastases, thoracic lymphadenopathy, and right upper chest wall mass (Fig 2). The patient has been receiving pembrolizumab for more than 2 years with close ophthalmologic monitoring but has not experienced any flare-up of his ocular sarcoidosis. He has experienced mild dermatologic immune-related adverse events (IRAEs), specifically an intermittent morbilliform rash managed with topical steroids as needed.

## Discussion

It is estimated that more than 15,000 patients die from cSCC annually in the United States.^2^ Fortunately, anti–PD-1 agents have greatly improved outcomes in patients with advanced and metastatic cSCC leading to high rates of objective responses, which tend to be durable.^3^ Unfortunately, clinical trials of ICI typically exclude patients receiving long-term systemic immunosuppression for AID or organ transplant, due to concerns for decreased efficacy and increased toxicity. In our patient with severe ocular sarcoidosis, there was major concern for vision loss with PD-1 inhibitor initiation. However, as our patient’s cSCC continued to progress with multiple cutaneous and lung metastases, the risk of death from cSCC far outweighed the risk of sarcoidosis exacerbation. Fortunately, our patient experienced durable benefit with ongoing impressive response for more than 2 years and without exacerbation of the underlying AID, demonstrating that the use of ICI may be safe in a subset of patients with AID. Indeed, several studies have recently shown feasibility of using ICIs in patients with AID or allografts, although close monitoring is warranted in these high-risk patients as they are likely to require aggressive management of IRAEs.^4^, ^5^, ^6^ However, it is important that clinical decisions regarding ICI use in patients at high risk of IRAEs are implemented after a thorough and personalized risk-benefit analysis in each patient’s unique clinical context.

## Conflicts of interest

Shailender Bhatia has received consulting or advisory role fees and honoraria from Bristol Myers Squibb and Incyte and his institution has received research funding from Bristol Myers Squibb, Merck, EMD Serono, Exicure, Incyte, Xencor, Checkmate Pharmaceuticals/Regeneron, 4SC, Seven and Eight Biopharmaceuticals, Amphivena Therapeutics, TriSalus Life Sciences, and Agenus. Mariam Totonchy is a speaker for Regeneron. Charlotte Read has no conflicts of interest to declare.
